# The microbiological and metabolic traits associated with pT3 colorectal cancer metastasis to lymph nodes

**DOI:** 10.3389/fphys.2025.1724429

**Published:** 2025-11-18

**Authors:** Lei Chen, Yiwen Zhang, Yilin Wang, Dianbo Cao, Jiandong Tai, Kaiyue Gao

**Affiliations:** 1 Department of Colorectal and anal surgery, General Surgery Center, The First Hospital of Jilin University, Jilin, China; 2 College of Chinese Medicine, Changchun University of Chinese Medicine, Changchun, China; 3 Department of Radiology, The First Hospital of Jilin University, Jilin, China

**Keywords:** colorectal cancer, lymph node metastasis, metabolite, gut microbiota, transcriptomes

## Abstract

**Introduction:**

Gut microbiota and metabolites play a crucial role in the progression of colorectal cancer. Over half of the CRC patients are at pT3 stage, the presence or absence of regional lymph node metastasis in pT3 patients significantly influences both treatment strategies and prognosis. However, the associations between these are not been revealed yet. It is crucial to gain a deeper insight into the mechanisms underlying the differences in gut microbiota and metabolites between pT3 CRCs with and without lymph node metastasis.

**Methods:**

We processed 16S rRNA gene sequencing and ultrahigh-performance liquid chromatography–mass spectrometry in 70 pT3NxM0 CRC patients. In addition, transcriptomic data from TCGA were retrieved to assess RNA expression differences between the two groups for a comprehensive comparison. Finally, correlation analyses of microbiota, metabolome and transcriptomic data were performed to identify meaningful connections and mechanisms underlying lymph node metastasis.

**Results:**

A total of 192 metabolites were different between the patients with and without lymph node metastasis; among these metabolites, 94 upregulated different metabolites were enriched in biological processes of tumor progression. The gut microbiota of lymph node positive CRCs is characterized by increased abundances of cancer progression, such as *Proteobacteria*. We identified 226 differentially expressed genes from TCGA-CRC cohort, among which the upregulated genes were mainly involved in pathways of cancer progression, proliferation, migration, and invasion, while downregulated genes were significantly enriched in pathways of tyrosine metabolism and immunity. The cross-correlation analysis showed that the altered metabolites and genes were enriched in neuroactive ligand receptor interaction pathway.

**Conclusion:**

Our study identified key microbiota and metabolites associated with lymph node metastasis in pT3 colorectal cancer, along with potential pathways and interactions implicated in the lymph node metastasis.

## 1 Introduction

Colorectal cancer (CRC) is one of the most common and high-risk malignant tumors in the world. Globally, the incidence and mortality rates of CRC rank third and second among malignant tumors, respectively. Each year, approximately 1.2 million cases are diagnosed, and more than 600,000 patients die from this disease (Mil et al., 2022; Bray et al., 2018). It’s worth noting that the incidence and mortality of CRC saw a rise in recently years due to changes in dietary habits (Chow et al., 2015), especially among younger individuals (Allen and Sears, 2019). The overall 5-year survival rate for CRC is around 60% according to related research (Siegel et al., 2017), with lymph node metastasis being a crucial factor in tumor staging and significantly affecting the prognosis of CRC patients (Lykke et al., 2019; Fortea-Sanchis et al., 2018; Amri et al., 2016). Numerous studies have reported that patients with lymph node metastasis tend to receive more aggressive treatment, poorer survival and higher recurrence rates compared to those with lymph node-negative (Böckelman et al., 2015; O’Connell et al., 2004; Jemal et al., 2008): CRC patients without lymph node metastasis have a 5-year overall survival rate as high as 80%–90%, whereas for those with metastasis, the rate is only 60%–68%. Notably, more than half of colorectal cancer patients are diagnosed at the pT3 stage (Foersch et al., 2022), making it the most prevalent pT stage among CRCs. Critically, lymph node metastasis status serves as a key determinant in therapeutic decision-making for pT3 CRC, particularly in determining the necessity of chemotherapy. Given its high prevalence and the pivotal role of nodal status in treatment stratification, we focused our investigation on pT3 CRC patients. This study aims to elucidate the mechanisms driving lymph node metastasis and identify potential biomarkers that could inform more personalized treatment strategies for this substantial patient population.

In recent years, with the deepening of research on microbiota and metabolites, an increasing amount of evidence has demonstrated its intricate connections between these factors and the occurrence and progression of tumors, as well as metastasis (Jia et al., 2021; Bergers and Fendt, 2021). For example, certain pathogenic bacteria, such as *Enterococcus faecalis (E. faecalis)* and *Streptococcus bovis (S. bovis)*, have been reported to play an important role in long-term chronic inflammation and further promote CRC development (Wang and Huycke, 2015). *F. nucleatum (Fusobacterium nucleatum)*, one of the most extensively studied pathogenic bacteria, has the ability to invade tumor cells, influence the epithelial-mesenchymal transition (EMT) in tumors and induce tumor metastasis to the liver or lungs (Chen S. et al., 2022; Yin et al., 2022; Zhang et al., 2022). Metabolic products of the gut microbiota serve as key mediators of the crosstalk between the gut microbial community and the human body and are closely associated with the development of cancers, including CRC. For instance, some metabolites produced by gut microbiota, such as bile acids (BAs), are linked to tumor progression (Jia et al., 2018). Besides, *Deoxycholic Acid* (DCA) were also reported as a tumor promoter in CRC (Ridlon and Bajaj, 2015). However, the metabolomics characteristics and underlying mechanism of colorectal cancer regional lymph node metastasis status has not been thoroughly explored until now.

In this study, we enrolled 70 pT3 colorectal adenocarcinoma patients with or without regional lymph node metastasis and collected their tumor surgical tissues. 16S rRNA gene sequencing and non-targeted Liquid chromatography-mass spectrometry (HP-LC-MS) approach were used to analyze their gut microbiota and metabolites. We compared metabolites and microbiota differences between lymph node negative and lymph node positive pT3 CRC patients. In addition, by integrating RNA-seq transcriptomics data of TCGA-CRC cohort from the TCGA (TCGA, PanCancer Atlas) database, we finally explore the connection between metabolites and gene expression and shedding light on the underlying mechanism of pT3 CRC metastasis to lymph nodes.

## 2 Materials and methods

### 2.1 Patient sample collection

**FIGURE 1:**
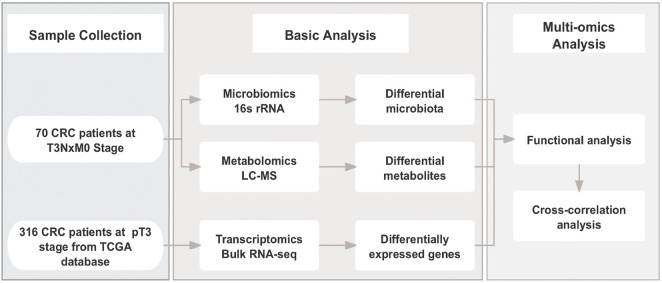
Overall experimental design and analysis workflow.

Human specimens were obtained from the Department of Biobank, Division of Clinical Research, The first hospital of Jilin University. A total of 70 patients with pT3NxM0 colorectal adenocarcinoma between January 2022 and July 2023 at The First Hospital of Jilin University, Jilin, China were enrolled in this study. They were divided into two groups based on whether or not they had lymph node metastasis: Lymph node negative (LN-Neg, n = 34) and Lymph node positive (LN-Pos, n = 36). The Ethical Committee of The First Hospital of Jilin University granted ethical approval for this observational retrospective research (approval number: 2023-535), and all patients provided written informed consent. The following demographic, clinical, and pathological data were collected: gender, age, Diabetes mellitus type 2 (T2DM), hypertension, location, differentiation grade, pathological types, neural invasion, vascular invasion and Body Mass Index (BMI). Fresh frozen tumor tissues from CRC patients were collected at surgery. The flow chart of this work is displayed in Figure 1.

### 2.2 16S rRNA gene sequencing data

The CTAB method was used for total genomic DNA extraction. Finally, DNA samples were diluted to 1 ng/L with sterile Deionized (DI) water.

Amplification of the 16S rRNA gene from different regions (16SV34) was performed using specific primers (314F-806R). All PCR reactions were processed using 15 μL of Phusion® High-Fidelity PCR Master Mix (New England Biolabs). Thermal cycling test were carried out by following steps: initial denaturation at 98 °C for 1 min, followed by 30 cycles of denaturation at 98 °C for 10 s, and annealing at 50 °C for 30 s. Finally, the samples were elongated at 72 °C for 30 s and 72 °C for 5 min. Mix the PCR product with an equal volume of 1X loading buffer (including SYBR Green) and perform gel electrophoresis on a 2% agarose gel. Mix the PCR products in equal density ratios. Then the PCR products were purified using the Qiagen Gel Extraction Kit (Qiagen, Germany). Libraries were constructed using the TruSeq® DNA PCR-Free Sample Preparation Kit (Illumina, United States). The constructed libraries were quantified using the Qubit@2.0 Fluorometer (Thermo Scientific) and the Agilent Bioanalyzer 2100 system. After qualitative assessment, libraries were pooled and sequenced using the Illumina NovaSeq 6000.

### 2.3 LC-MS data acquisition

Take 100 mg of the sample in an EP tube, add 500 μL of 80% methanol aqueous solution. Incubate the samples on ice for 5 min, then centrifuge at 4 °C at 15,000 × g for 20 min. Take a certain amount of supernatant and dilute with LC-MS grade water until the methanol content is 53%. Centrifuge at 15,000 × g and 4 °C for 20 min. Finally, collect the supernatant and inject it into the LC-MS system for analysis. Take equal volumes from each sample and mix as quality control (QC) samples.

Vanquish UHPLC system (Thermo Fisher, Germany) combined with an Orbitrap Q ExactiveTM HF-X mass spectrometer (Thermo Fisher, Germany) was used for UHPLC-MS spectrometry analysis. Prepared samples were injected onto a Hypersil Gold column (100 × 2.1 mm, 1.9 µm) at a flow rate of 0.2 mL/min with a linear gradient of 12 min. The positive polarity eluents were eluent A (0.1% formic acid (FA) in water) and eluent B (methanol). The negative polarity eluents were eluent A (5 mM ammonium acetate, pH 9.0) and eluent B (methanol). The gradient elution program is as follows: 2% B, 1.5 min; 2%-85% B, 3.0 min; 85%-100% B, 14.0 min; 100%-2% B, 10.1 min; 2% B, 12 min. For mass spectrometry, the following parameters were set by Q Exactive TM HF mass spectrometer: i) polarity mode = positive/negative; ii) spray voltage = 3.5 kV; iii) sheath gas flow rate = 35 psi; iv) was operated in with; v) capillary temperature = 320 °C; vi) aux gas flowrate = 10L/min; vii) S-lens RF level = 60; viii) Aux gas heater temperature = 350 °C.

### 2.4 Transcriptomic cohort and data sources

Gene expression profiles and clinical data of CRC patients were downloaded from the TCGA (TCGA, PanCancer Atlas) via the cBioPortal (http://www.cbioportal.org/). Totally 633 colorectal cancer cases with clinical information were downloaded, and finally 316 patients included after screening: 1)patients with stage pT3 were selected, leaving 447 cases, 2) patients with unknown lymph node status were excluded (445 patients left); 3) patients lacking follow-up data, having less than 3 months of follow-up, or missing time of death data, as well as those with too short a survival time (0 or 1 day), were excluded (383 patients left), 4) patients with unknown primary site/connective state were excluded (381 patients left), 5) patients without RNA-seq data were excluded (377 patients left), 6) advanced patients were excluded (316 patients left).

### 2.5 Data analysis

#### 2.5.1 16S rRNA gene sequencing data analysis

Paired-end reads were generated based on the barcodes of each sample and assigned to the respective samples, which were then merged using the FLASH (Fast Length Adjustment of SHort reads) software. High-quality filtering of the raw tags was conducted to acquire clean tags using the fastp (Version 0.23.1) software (Bokulich et al., 2013). The tags were aligned against the Silva database (16S) (https://www.arb-silva.de/) to identify chimeric sequences using the UCHIME Algorithm (http://www.drive5.com/usearch/manual/uchime_algo.html) (Edgar et al., 2011). After chimeric sequences were eliminated, the remaining high-quality non-chimeric tags were retained as effective tags for further analysis.

These effective tags were subjected to denoise using the DADA2 (Callahan et al., 2016) or deblur plugin in the QIIME2 software (version QIIME2-202202) to acquire the initial amplicon sequence variants (ASVs), with DADA2 being the default choice. Species annotation and statistical analysis at different taxonomic levels (kingdom, phylum, class, order, family, genus, species) were performed by comparison with the Silva138.1 database using the QIIME2 software.

Alpha diversity was analyzed to assess community richness and diversity using the Chao1 and Shannon indices, while beta diversity analysis, performed via qiime2, was used to evaluate differences in species complexity between samples. Prior to clustering analysis, principal component analysis (PCA) was conducted on the raw non-dimensional variables using the FactoMineR and ggplot2 packages in R software (Version 2.15.3). Finally, MetaStat analysis was performed using the ComplexHeatmap R package to detect community structure differences, with significance set at Q value <0.05.

#### 2.5.2 LC-MS-based metabolomics data analysis

Raw mass spectrometric data of this research have been uploaded to MetaboLights (https://www.ebi.ac.uk/metabolights/), they can be accessed using the following information: deposit ID: MTBLS11418. The raw data files from UHPLC-MS/MS platform were processed by using Compound Discoverer 3.3 (CD3.3, Thermo Fisher). Initial screening was conducted for retention time, mass-to-charge ratio, and other relevant features of each metabolite. To improve identification precision, peak alignment was standardized across samples with a retention time deviation of 0.2 min and a mass deviation of 5 PPM. Peak intensities were then normalized against the total spectral intensity. The normalized data were utilized for molecular formula prediction, considering additive ions, molecular ion peaks, and fragment ions.

Subsequent peak matching against the mzCloud (https://www.mzcloud.org/), mzVault, and MassList databases ensured accurate qualitative and semi-quantitative analysis. Data processing was conducted using R software (version 3.4.3), Python (version 2.7.6), and CentOS (version 6.6). For non-normally distributed data, area normalization was applied to transform the data to a normal distribution. Metabolite annotation was facilitated using the KEGG (https://www.genome.jp/kegg/pathway.html), HMDB (https://hmdb.ca/metabolites), and LIPIDMaps (http://www.lipidmaps.org/) databases.

Multivariate statistical analysis was performed using MetaX (a flexible and comprehensive software for processing metabolomics data) for data preprocessing, followed by principal component analysis (PCA) and partial least square discriminant analysis (PLS-DA), which were conducted using SIMCA 14.1 (Umetrics, Sweden). Univariate analysis (t-test) calculated the statistical significance (P value) of each metabolite between groups using a T-test, along with fold change (FC) values. Metabolites were identified as differentially expressed if they exhibited a VIP score >1, P value <0.05, and a fold change (FC) ≥2 or ≤0.5.

A volcano plot was created using the ggplot2 package in R, integrating VIP values, log2 (Fold Change), and log10 (P value) to identify metabolites of interest. Clustering heatmaps were generated using the Pheatmap package in R, with metabolite data normalized by z-scores. The KEGG database was referenced to explore the functions and metabolic pathways associated with the metabolites. Metabolic pathway enrichment analysis was performed separately for upregulated and downregulated metabolites, and a pathway was considered enriched when the condition x/n > y/N was met. A metabolic pathway was deemed significantly enriched if P value <0.05.

#### 2.5.3 Transcriptome analyses

R package DESeq2 (Love et al., 2014) was introduced to identify the differentially expressed genes between comparisons by using the readcount matrix downloaded from TCGA database via cBioPortal. And the threshold is set as: | log2 (Fold Change) | >1 and P value <0.05 to filter out the credible DEGs.

The volcano plot was crafted with the R package (ggplot2), and clustering heatmaps were generated with the ComplexHeatmap package in R. To better understand the possible function of DEGs between two groups, the ClusterProfiler package in R was employed to do the KEGG enrichment analysis on upregulated or downregulated DEGs separately with threshold P value <0.05 for significantly enriched pathway.

#### 2.5.4 Interaction analysis between different omics

An interaction analysis between different omics datasets was conducted to explore the overall molecular characteristics and key biomarkers associated with lymph node metastasis. Metabolomics data served as a connecting point to integrate the transcriptome and microbiome data. Specifically, the functional relationship between the transcriptome and metabolome was investigated through a comparative KEGG pathway enrichment analysis of DEGs and differential metabolites. The R package ggplot2 was used to visualize shared pathways between the metabolic pathway enrichment results and gene expression pathway enrichment results, highlighting common pathways between metabolic and gene expression data.

Additionally, the R package corrplot was employed to identify significant correlations between differential metabolites and differential community structure, with a significance threshold of P value <0.05.

## 3 Result

### 3.1 Patient characteristics

The clinical characteristics of pT3 CRCs in lymph node negative (LN-Neg, n = 34) and lymph node positive (LN-Pos, n = 36) cohorts are summarized in Table 1. Among the LN-Neg group, 23 (67.6%) were male and 11 (32.4%) were female, with a median BMI of 23.5, compare with 24 (66.7%) males and 12 (13.3%) females for LN-Pos group, which had a median BMI of 24.5. The age distribution was similar between these two groups: both groups had roughly equal numbers of patients under and over 65 years old (n = 15 and n = 19 for LN-Neg, n = 19 and n = 19 for LN-Pos). Additionally, 31 patients (91.2%) in LN-Neg cohort and 28 (77.8%) in LN-Pos cohort had no hypertension respectively. The rectum was the most common tumor location (n = 23, 67.6% and n = 17, 47.5%) in both two cohorts. Medium differentiation accounted for 88.2% (n = 30) and 58.3% (n = 21) in two group, separately, indicating that Poor differentiation is associated with a higher likelihood of lymph node metastasis. In terms of pathological type, the LN-Neg group included anabrotic (n = 14, 41.2%), tumeur (n = 17, 50.0%) and infiltrating (n = 3, 8.8%) cases, whereas the LN-Pos group had 23, 10 and 3 cases, respectively. Furthermore, 67.6% of LN-Neg patients and 55.6% of LN-Pos patients had no neural invasion. However, vascular invasion was much more common in LN-Pos patients (n = 30, 83.3%) compared to LN-Neg patients (n = 6, 17.6%). Overall, the clinical characteristics were comparable between the lymph node negative and lymph node positive cohorts.

**TABLE 1:** Patient characteristics.

Characteristics	LN_Neg	LN_Pos	P-value
	(N = 34)	(N = 36)	
Gender			1
Male	23 (67.6%)	24 (66.7%)	
Female	11 (32.4%)	12 (33.3%)	
Age			0.984
	15 (44.1%)	17 (47.2%)	
≥65 years	19 (55.9%)	19 (52.8%)	
T2DM			0.676
No	26 (76.5%)	30 (83.3%)	
Yes	8 (23.5%)	6 (16.7%)	
Hypertension			0.226
No	31 (91.2%)	28 (77.8%)	
Yes	3 (8.8%)	8 (22.2%)	
Location			0.0742
Rectum	23 (67.6%)	17 (47.2%)	
Left-sided	5 (14.7%)	14 (38.9%)	
Right-sided	6 (17.6%)	5 (13.9%)	
Differentiation_grade			0.011
Low	4 (11.8%)	15 (41.7%)	
Medium	30 (88.2%)	21 (58.3%)	
Pathological_types			0.139
Anabrotic	14 (41.2%)	23 (63.9%)	
Tumeur	17 (50.0%)	10 (27.8%)	
Infiltrating	3 (8.8%)	3 (8.3%)	
Neural_invasion			0.428
No	23 (67.6%)	20 (55.6%)	
Yes	11 (32.4%)	16 (44.4%)	
Vascular_invasion			<0.001
No	28 (82.4%)	6 (16.7%)	
Yes	6 (17.6%)	30 (83.3%)	
BMI			0.332
Mean (SD)	23.5 (5.43)	24.5 (3.59)	
Median [Min, Max]	22.6 [17.7, 49.3]	24.4 [15.9, 32.3]	

### 3.2 Metabolic analysis between lymph node negative and lymph node positive CRC patients

**FIGURE 2:**
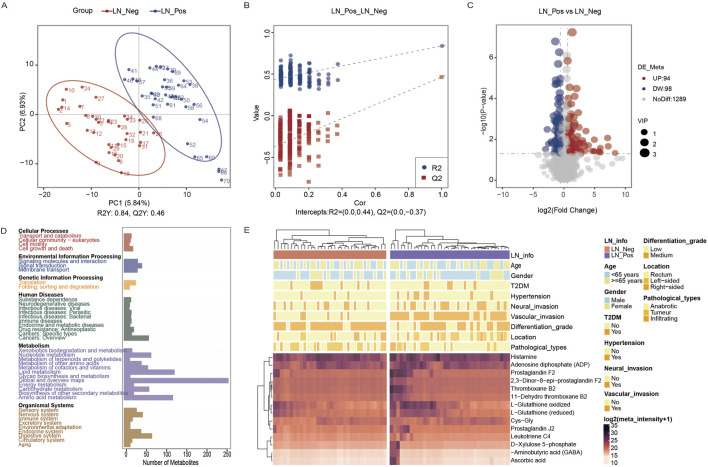
Metabolic analysis. **(A)** PLS-DA plot of the LN_Neg and LN_Pos group. **(B)** Validation of the PLS-DA model. **(C)** Volcano plot of different metabolites between the LN_Neg and LN_Pos groups. **(D)** KEGG pathway annotations of all metabolites identified. **(E)** Heatmap of 14 differential metabolites participated in these significant enriched pathways. Four metabolites that contributed in these significantly enriched KEGG pathways.

In this study, a non-targeted metabolomics analysis was conducted based on LC-MS technology. PLS-DA, which offers superior discrimination power compared to PCA (Yu L. et al., 2021), was applied to analyze the metabolic profiles based on class information. The significant clustering observed in the PLS-DA model highlights a clear separation between lymph node-negative and lymph node-positive CRC patients (Figure 2A). A permutation test was conducted to evaluate the model’s quality. The model demonstrated high interpretability with R^2^Y = 0.84 and no signs of overfitting, as all permutation results were inferior to the original model (Figure 2B).

A total of 1040 metabolites in ESI + mode and 441 metabolites in ESI- mode were identified between lymph node negative and lymph node positive CRC patients (Supplementary Table S1). After annotation to the KO (KEGG Orthology) database, these metabolites were found to be involved in 6 main pathways including Cellular Processes, Environmental Information Processing, Genetic Information Processing, Human Diseases, Metabolism and Organismal Systems. Notably, the metabolites were primarily associated with in amino acid metabolism, including pathways such as Global and overview maps, Metabolism of cofactors and vitamins, and Amino acid metabolism (Figure 2D). Based on variable importance in the projection (VIP) values >1, fold change (FC) ≧2 or ≦0.5, and P value <0.05, 192 differential metabolites were identified (Supplementary Table S2), with 94 upregulated and 98 downregulated metabolites (Figure 2C). These metabolites were selected as references for further analyses.

KEGG enrichment analysis was then performed separately on 94 upregulated and 98 downregulated differential metabolites. Pathways were considered significantly enriched if P value <0.05, therefore 8 pathways were identified for upregulated metabolites: thyroid hormone synthesis, serotonergic synapse, arachidonic acid metabolism, asthma, neuroactive ligand-receptor interaction, glutathione metabolism, Fc epsilon RI signaling pathway and synaptic vesicle cycle (Table 2). 14 differential metabolites [Histamine, Adenosine diphosphate (ADP), Prostaglandin F2, 2,3-Dinor-8-epi-prostaglandin F2, Thromboxane B2, 11-Dehydro thromboxane B2, L-Glutathione oxidized, L-Glutathione (reduced), Cys-Gly, Prostaglandin J2, Leukotriene C4, D-Xylulose 5-phosphate, Aminobutyric acid (GABA), Ascorbic acid] participated in these significant enriched pathways. And their distribution between lymph node-negative group and lymph node-positive group was shown in the heatmap (Figure 2E). On the other hand, no significantly enriched pathways were found in downregulated metabolites.

**TABLE 2:** The KEGG pathways enrichment analysis of differential metabolites.

ID	Description	GeneRatio	BgRatio	pvalue	p.adjust	qvalue	geneID	Count
map04918	Thyroid hormone synthesis	3/53	4/379	0.0015395	0.1016069	0.0939904	Com_939_pos/Com_10233_pos/Com_6189_neg	3
map04726	Serotonergic synapse	5/53	16/379	0.0044576	0.1050836	0.0972065	Com_14876_neg/Com_14916_neg/Com_10942_neg/Com_14878_neg/Com_12560_neg	5
map00590	Arachidonic acid metabolism	6/53	23/379	0.0047765	0.1050836	0.0972065	Com_14876_neg/Com_14916_neg/Com_10942_neg/Com_12115_neg/Com_14878_neg/Com_12560_neg	6
map05310	Asthma	2/53	3/379	0.016192	0.2308453	0.2135411	Com_14876_neg/Com_478_pos	2
map04080	Neuroactive ligand-receptor interaction	5/53	22/379	0.0192844	0.2308453	0.2135411	Com_14876_neg/Com_478_pos/Com_2359_pos/Com_4675_neg/Com_12560_neg	5
map00480	Glutathione metabolism	4/53	15/379	0.0209859	0.2308453	0.2135411	Com_20058_pos/Com_939_pos/Com_10233_pos/Com_2733_neg	4
map04664	Fc epsilon RI signaling pathway	2/53	4/379	0.0308444	0.2908188	0.269019	Com_14876_neg/Com_478_pos	2
map04721	Synaptic vesicle cycle	2/53	5/379	0.0489744	0.3602289	0.3332261	Com_478_pos/Com_4675_neg	2

### 3.3 Microbiota analysis between lymph node negative and lymph node positive CRC patients

**FIGURE 3:**
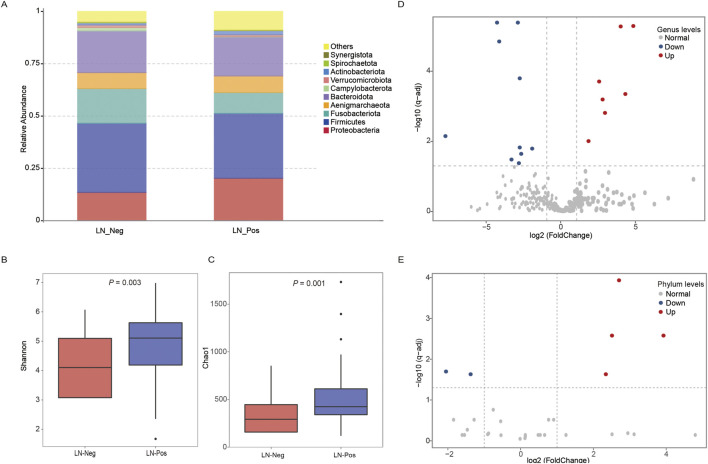
Microbiota analysis. **(A)** Taxonomic proportions according to compositions at the phylum level. **(B)** Shannon diversity index of LN_Neg and LN_Pos group. **(B)** Chao 1 index of LN_Neg and LN_Pos group. **(D)** Volcano plot of differential microbiota annotated at the genus level between the LN_Neg and LN_Pos groups. **(E)** Volcano plot of differential microbiota annotated at the phylum levels level between the LN_Neg and LN_Pos group.

We examined the compositional differences in the gut microbiota at the phylum levels between the two groups (Figure 3A). *Proteobacteria* were enriched in lymph node positive patients, whereas lymph node negative patients exhibited significantly higher abundances of *Fusobacteriota*.

Alpha diversity, which measures the number and proportion of microbial species within a sample (species evenness and richness) (Jiang et al., 2018), was analyzed in both groups. The Shannon diversity index and Chao 1 index revealed significant differences between the two groups (P = 0.003 and P = 0.001, respectively; Figures 3B,C), indicating that the gut microbiome was strongly associated with lymph node status.

To future explore these differences, we used the MetaStat method to perform hypothesis testing on species abundance data between groups, identifying significant differential microbial communities with Q value <0.05. Volcano plot (Figure 3E) displayed 6 differential microbiotas at Phylum levels, with 4 upregulated and 2 downregulated. Similarly, 8 upregulated and 10 downregulated differential microbial communities were identified at Genus levels (Figure 3D). Details of these the microbiota in this study and differential microbial communities are provided in Supplementary Tables S3, S4 separately.

### 3.4 Transcriptome analyses between lymph node negative and lymph node positive CRC patients

To further investigate the molecular mechanisms related to lymph node status, transcriptomics data of CRC patients were downloaded from the TCGA PanCancer Atlas through the cBioPortal (http://www.cbioportal.org/). A total of 316 cases including 187 lymph node negative and 129 lymph node positive patients, were analyzed after screening. The clinical features of these two groups were summarized in Supplementary Table S5.

**FIGURE 4:**
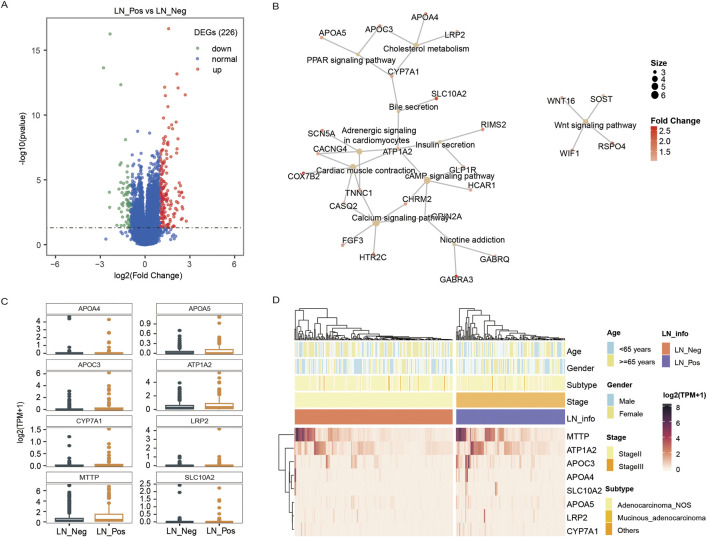
Transcriptomic analysis. **(A)** The volcano plot displays an overview of the differential expressed genes (DEGs) between the LN_Neg and LN_Pos group. **(B)** The fat metabolism KEGG pathways of DEGs. **(C)** The expression differences of genes contributed in fat metabolism KEGG pathway between LN_Neg and LN_Pos group. **(D)** Heatmap showing the differentially expressed genes involved in fat metabolism KEGG pathway.

**FIGURE 5:**
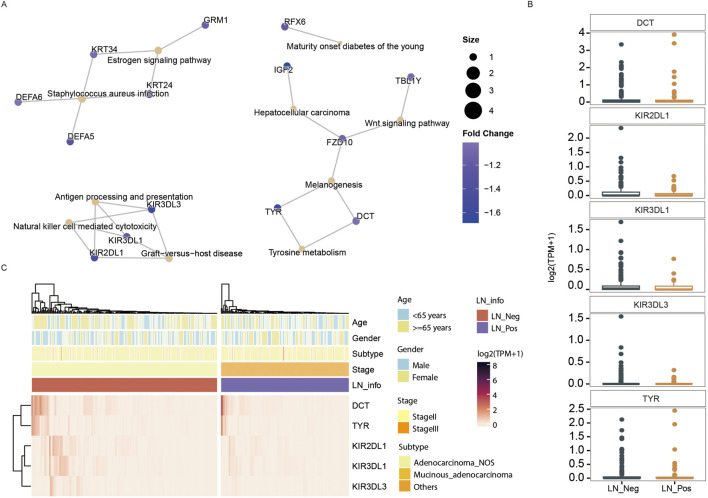
Transcriptomic analysis. **(A)** The downregulated KEGG pathways of DEGs. **(B)** The expression differences of genes contributed in downregulated KEGG pathway between LN_Neg and LN_Pos group. **(C)** Heatmap showing the differentially expressed genes involved in downregulated KEGG pathway.

We identified 226 differentially expressed genes (DEGs), with 167 genes upregulated and 59 genes downregulated between the two groups (Figure 4A; Supplementary Table S6). Subsequently, KEGG enrichment analysis was performed separately on the upregulated genes and downregulated genes to explore pathways associated with lymph node status. As shown in Table 3, the upregulated genes were significantly enriched in pathways related to fat metabolism including cholesterol metabolism, PPAR signaling pathway, bile secretion and fat digestion and absorption (Figure 4B). Meanwhile, downregulated genes (Table 4) were significantly enriched in pathways associated with tyrosine metabolism and immunity, such as antigen processing and presentation and natural killer cell-mediated cytotoxicity (Figure 5A).

**TABLE 3:** The KEGG pathways enrichment analysis of differential upregulated genes.

ID	Description	GeneRatio	BgRatio	pvalue	p.adjust	qvalue	geneID	Count
hsa04260	Cardiac muscle contraction	5/56	82/7624	0.000316	0.024233	0.02232	ATP1A2/CACNG4/CASQ2/COX7B2/TNNC1	5
hsa04979	Cholesterol metabolism	4/56	49/7624	0.000433	0.024233	0.02232	APOA4/APOC3/CYP7A1/LRP2	4
hsa05033	Nicotine addiction	3/56	40/7624	0.00306	0.114227	0.105209	GABRA3/GABRQ/GRIN2A	3
hsa04020	Calcium signaling pathway	6/56	238/7624	0.007653	0.214274	0.197357	CASQ2/CHRM2/FGF3/GRIN2A/HTR2C/TNNC1	6
hsa03320	PPAR signaling pathway	3/56	74/7624	0.016847	0.333399	0.307078	APOA5/APOC3/CYP7A1	3
hsa04024	cAMP signaling pathway	5/56	220/7624	0.022151	0.333399	0.307078	ATP1A2/CHRM2/GLP1R/GRIN2A/HCAR1	5
hsa04261	Adrenergic signaling in cardiomyocytes	4/56	149/7624	0.023466	0.333399	0.307078	ATP1A2/CACNG4/SCN5A/TNNC1	4
hsa04911	Insulin secretion	3/56	86/7624	0.025011	0.333399	0.307078	ATP1A2/GLP1R/RIMS2	3
hsa04976	Bile secretion	3/56	89/7624	0.027331	0.333399	0.307078	ATP1A2/CYP7A1/SLC10A2	3
hsa04310	Wnt signaling pathway	4/56	167/7624	0.033781	0.333399	0.307078	RSPO4/SOST/WIF1/WNT16	4
hsa04975	Fat digestion and absorption	2/56	40/7624	0.034589	0.333399	0.307078	APOA4/MTTP	2
hsa05202	Transcriptional misregulation in cancer	4/56	170/7624	0.035721	0.333399	0.307078	PAX7/SSX1/WNT16/ZBTB16	4
hsa04360	Axon guidance	4/56	180/7624	0.042652	0.354001	0.326053	EPHA6/EPHA8/SEMA3D/SEMA3E	4
hsa04080	Neuroactive ligand-receptor interaction	6/56	354/7624	0.04425	0.354001	0.326053	CHRM2/GABRA3/GABRQ/GLP1R/GRIN2A/HTR2C	6

**TABLE 4:** The KEGG pathways enrichment analysis of differential downregulated genes.

ID	Description	GeneRatio	BgRatio	pvalue	p.adjust	qvalue	geneID	Count
hsa05150	*Staphylococcus aureus* infection	4/22	89/7624	0.0001081	0.0054388	0.0051179	DEFA5/DEFA6/KRT24/KRT34	4
hsa05332	Graft-versus-host disease	3/22	38/7624	0.0001648	0.0054388	0.0051179	KIR2DL1/KIR3DL1/KIR3DL3	3
hsa04612	Antigen processing and presentation	3/22	69/7624	0.000966	0.0212516	0.0199975	KIR2DL1/KIR3DL1/KIR3DL3	3
hsa04916	Melanogenesis	3/22	101/7624	0.0028942	0.0477547	0.0449367	DCT/FZD10/TYR	3
hsa00350	Tyrosine metabolism	2/22	36/7624	0.0047192	0.056756	0.0534068	DCT/TYR	2
hsa04650	Natural killer cell mediated cytotoxicity	3/22	124/7624	0.0051596	0.056756	0.0534068	KIR2DL1/KIR3DL1/KIR3DL3	3
hsa04915	Estrogen signaling pathway	3/22	137/7624	0.0068076	0.064186	0.0603983	GRM1/KRT24/KRT34	3

The expression of all DEGs contributing to the significant enriched pathways between the two groups is summarized in Table 3. To specify, eight genes were closely involved in fat metabolism: APOA4, APOA5, APOC3, ATP1A2, CYP7A1, LRP2, MTTP and SLC10A2. And the differential distributions of these DEGs between the lymph node negative and positive groups are depicted in Figures 4C,D. Additionally, five downregulated genes involved in tyrosine metabolism and immunity pathway were identified: DCT, KIR2DL1, KIR3DL1, KIR3DL3 and TYR (Figure 5A), with their expression differences shown in Figures 5B,C.

### 3.5 Cross-correlation analysis among the microbiota and metabolites and transcriptome

**FIGURE 6:**
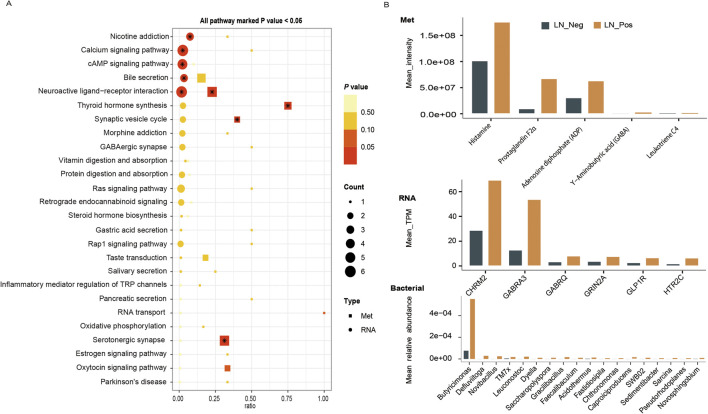
Cross-Correlation Analysis. **(A)** The KEGG pathway enrichment analysis of transcriptome DEGs and differential metabolites between the LN_Neg and LN_Pos group. **(B)** Mean values of microbiota, metabolites and transcriptome that involved in the neuroactive ligand-receptor interaction pathway.

To investigate overall molecular characteristics of lymph node metastasis and find out the key biomarkers for lymph node metastasis, the relationships between different omics were explored through interaction analysis by using metabolomics data as the link. We explore the functional relationship between differentially expressed RNA genes transcriptome and metabolome altered metabolites by conducting a comparative KEGG pathway enrichment analysis of transcriptome DEGs and differential metabolites between the two groups. As shown in Figure 6A, the neuroactive ligand-receptor interaction pathway was significantly upregulated in both the transcriptome and metabolome. We further looked into the specific metabolites and DEGs involved in the neuroactive ligand-receptor interaction pathway. Five differential metabolites were enrolled in this pathway, including Adenosine diphosphate (ADP), Histamine, Leukotriene C4, Prostaglandin F2α and Υ-Aminobutyric acid (GABA) (Figure 6B meta). Meanwhile, six DEGs contributed to this pathway, such as HTR2C, GRIN2A, GLP1R, GABRQ, GABRA3, CHRM2 (Figure 6B RNA).

**FIGURE 7:**
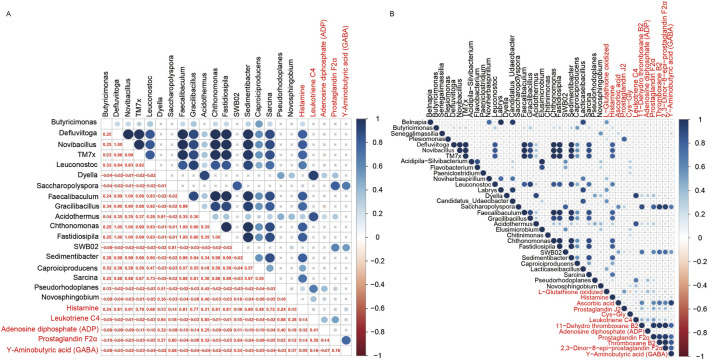
Cross-correlation analysis between microbiota and metabolites. **(A)** Bubble diagram of the correlation between the microbiota annotated at the genus level and metabolites that involved in the neuroactive ligand-receptor interaction pathway. **(B)** Bubble diagram of the correlation between the microbiotas annotated at the genus level and metabolites that significantly enriched in the KEGG pathways.

On the other hand, pearson correlation analysis was conducted on the quantification data of differential microbial communities at genus level and differential metabolites to figure out the association between microbiomics and metabolome (Figure 7B; Supplementary Table S7). To study the differential microbial communities at genus level, which are also related to the neuroactive ligand-receptor interaction pathway, a correlation analysis between these microbial communities and the differential metabolites enriched in this pathway was performed. Eighteen differential microbiotas were significantly associated to the differential metabolites that involved in the neuroactive ligand-receptor interaction pathway (Figure 7A), such as *Acidothermus, Butyricimonas, Caproiciproducens, Chthonomonas, Defluviitoga, Dyella, Faecalibaculum, Fastidiosipila, Gracilibacillus, Leuconostoc, Novibacillus, Novosphingobium, Pseudorhodoplanes, Sarcina, Sedimentibacter* and *TM7x* (Supplementary Table S8). And their mean relative abundances were illustrated in Figure 6B bacterial, with genus Butyricimonas showing particularly high abundance. In the meantime, as shown in Figure 7A, Histamine exhibited significantly correlations with various microbiota, and Leukotriene was significantly correlated with *Acidothermus*. Additionally, Prostaglandin F2α and Υ-Aminobutyric acid (GABA) were significantly correlated with *Saccharopolyspora*.

## 4 Discussion

The burgeoning field of metabolomics research have provided more and more evidence that microbial communities are intricately intertwined with the genesis and progression of cancer. Therefore, metabolomics has emerged as a useful tool for identifying novel diagnostic and prognostic biomarkers, as well as developing new therapeutic targets for a variety of diseases (Mato et al., 2019; Patel and Ahmed, 2015; Perakakis et al., 2020). The large bowel is the part of the human body with the highest amount of bacteria, and the microorganisms in it can interact with colorectal mucosal epithelial cells to regulate the basic physiological activities of the host, including energy intake, metabolic regulation and immune homeostasis (Li et al., 2022).

To profile the microbiological and metabolic characteristics and understand the underlying molecular mechanisms of lymph node metastasis in pT3 CRC patients, 16S rRNA sequencing and untargeted metabolomics was utilized to detect differential microbiota and metabolites in the tumor tissues of Chinese pT3 colorectal adenocarcinoma patients with or without lymph node metastasis. Additionally, we further downloaded the pT3 CRC transcriptomic data from the TCGA database to identify lymph node metastasis-related differentially expressed genes. Our findings revealed that: (1) pT3 CRCs with lymph node positivity exhibited higher levels metabolites involved in pathway related to pro-inflammatory and tumor-promoting; (2) distinct microbiological characteristics between pT3 CRCs with or without lymph node metastasis; (3) significant upregulation of gene expressions associated with cancer progression, proliferation, migration, and invasion in pT3 CRCs with node-positive; (4) both differential RNA expression and metabolites were significantly enriched in neuroactive ligand-receptor interaction pathway in lymph node positive pT3 CRC patients. These results suggested that the metabolites affect importantly in lymph node metastasis through neuroactive ligand-receptor interaction pathway.

With regard to the metabolome, many studies have demonstrated that metabolite profiles are associated with CRC early diagnosis by using plasma and fecal samples (Sun et al., 2024; Chen F. et al., 2022; Coker et al., 2022; Yang et al., 2022), but few studies focused on pT3NxM0 CRC patient or explore their association with lymph node status. In our study, the metabolomics analysis indicated that pT3 CRC patients with lymph nodes positive had distinct metabolic characteristics compared to those without. Further KEGG enrichment analysis revealed that these metabolites enriched in biological process that promote inflammation and tumor proliferation, such as thyroid hormone synthesis, arachidonic acid metabolism and glutathione metabolism. Ma et al. (2023) reported that thyroid hormone are key regulators of energy metabolism and homeostasis, influencing processes like protein synthesis, glycogen breakdown and synthesis, and the oxidation of fatty acids and the synthesis and degradation of cholesterol (Ma et al., 2023). Our study also found upregulation of the thyroid hormone pathway in lymph node positive cohort, suggesting heightened cellular activity among these patients. In addition, glutathione metabolism plays a crucial role in tumor progression as it not only supports mitochondrial oxidative phosphorylation but also provides metabolic intermediates for the TCA cycle, glutathione synthesis, and non-essential amino acid (NEAA) synthesis, while simultaneously generating NADPH (Yoo et al., 2020). The upregulated differential metabolites of lymph node positive cohort were significantly enriched in this pathway in our result demonstrated that tumor progression is more advanced in these patients compared with these without LNM metastasis.

Microbiota are increasingly recognized as key influencers of cancer development and prognosis. Thompson KJ et al. found that the abundance of *Proteobacteria* is relatively high in breast cancer tumor tissues, while *Actinobacteria* are more abundant in the adjacent healthy tissues (Thompson et al., 2017). Similarly, we observed an enrichment of *Proteobacteria* in lymph node positive CRC patients, whereas *Fusobacteriota* were more prevalent in lymph node-negative patients. Moreover, Alexander JL et al. reported that *Faecalibacterium prausnitzii* and *Ruminococcus gnavus* linked to poorer disease-free survival outcomes for CRC patients following resection (Alexander et al., 2023).

In terms of transcriptome, RNA expression characteristics of pT3 CRCs with lymph node metastasis were different from patients without lymph node metastasis. KEGG enrichment analysis showed that upregulated genes in lymph node-positive patients were predominantly involved in fat metabolism, including cholesterol metabolism, PPAR signaling pathway, bile secretion and fat digestion and absorption. Metabolic reprogramming is a hallmark of cancer progression (Hakimi et al., 2016). PPARs (Peroxisome Proliferator-Activated Receptors) are a class of nuclear receptors that play a key role in various aspects such as lipid metabolism, energy balance, inflammatory responses, and cell differentiation within the cell. The PPAR signaling pathway influences the metabolic reprogramming, proliferation, migration, and invasion of cancer cells to further promote tumor growth (Li Y. et al., 2024). Similarly, altered cholesterol metabolism can generate oncogenic metabolites and suppress anti-tumor immune responses, which may support the survival and migration of cancer cells, a finding consistent with studies linking metabolic dysregulation to cancer progress (Huang et al., 2020). Our findings explained from transcriptome level that patients with lymph node metastasis have a higher degree of tumor invasion. Cross-correlation KEGG enrichment analysis of untargeted metabolomics combined with TCGA transcriptomic data suggested upregulation of the neuroactive ligand-receptor interaction pathway in lymph node positive cohort. This pathway, which encompasses all receptors and ligands involved in signaling inside and outside the cell, has been implicated in various diseases, including cancer. Ji X et al. reported that neuroactive ligand receptor interaction pathway was significantly associated with lung cancer risk (Ji et al., 2018). Besides, there are also some researches about prognostic value of neuroactive ligand receptor interaction pathway. Yang Y et al. reported that neuroactive ligand receptor interaction pathway was the independent prognostic factor in colon adenocarcinoma (COAD) and targeted genes in this pathway can increase treatment response to immunotherapy (Yang et al., 2023). Similarly, Yu J found higher TMB (tumor mutation burden) was correlated with better survival outcome in gastric cancer patients, and TMB-high group were also associated with neuroactive ligand-receptor interaction pathway (Yu J. et al., 2021).

This study has several limitations that should be acknowledged. First, the relatively small patient cohort limited our ability to explore the impact of type 2 diabetes (T2DM)—a recognized independent risk factor for colorectal cancer progression and metastasis—on lymph node metastasis in our dataset (Ottaviano et al., 2020; Li J. et al., 2024). Second, while our analysis identified metabolic alterations associated with lymph node metastasis, transcriptomic validation was not performed in the same patient cohort. Instead, we relied on RNA-seq data from the public TCGA-CRC dataset. Third, due to the lack of long-term follow-up information, survival analysis could not be conducted. This is particularly relevant given that lymph node metastasis is a well-established prognostic factor in colorectal cancer, with studies showing decreased survival as the number of metastatic lymph nodes increases (Parsons et al., 2011). Although our results suggest that patients with lymph node metastasis exhibit elevated levels of tumor-promoting metabolites—implying a potentially worse prognosis—this association requires further confirmation in future studies with comprehensive clinical outcome data.

Comparing prognostic differences of patients would provide direct insight into how differential microbiota and metabolites influence patient prognosis. Therefore, further studies are still needed to validate our protocol.

## 5 Conclusion

Patients with pT3 colorectal cancer metastasis to lymph nodes display unique microbiological profiles, significantly enriched metabolites that involved in pro-inflammatory responses and tumor promotion and a notable upregulation of gene expression which are associated with cancer progression, proliferation, migration, and invasion. Cross-correlation KEGG enrichment analysis of differential RNA expression and metabolite profiles show significant enrichment within the neuroactive ligand-receptor interaction (NLRI) pathway. Collectively, our results suggest that metabolites play a pivotal role in facilitating lymph node metastasis, potentially through modulation of the NLRI pathway.

## Data Availability

The datasets presented in this study can be found in online repositories. The names of the repository/repositories and accession number(s) can be found in the article/Supplementary Material.
